# “Don’t rush into thinking of walking again”: Patient views of treatment and disability following an open tibia fracture in Malawi

**DOI:** 10.12688/wellcomeopenres.18063.1

**Published:** 2022-08-09

**Authors:** Alexander Thomas Schade, Wakumanya Sibande, Moses Kumwenda, Nicola Desmond, Linda Chokotho, Eleni Karasouli, Andrew Metcalfe, William J. Harrison

**Affiliations:** 1 Public Health, Malawi-Liverpool-Wellcome Trust, Blantyre, Malawi; 2 Clinical Trials Unit, University of Warwick, Coventry, UK; 3 Trauma and Orthopaedics, Queen Elizabeth Central Hospital, Blantyre, Malawi; 4 Liverpool School of Tropical Medicine, Liverpool, UK; 5 AO Alliance, Davos, Switzerland

**Keywords:** Road traffic injury; open fractures; disability; function; low-income country

## Abstract

**Background:** Open tibia fractures are a common injury following road traffic accidents in Malawi and can lead to long term disability. Very little is known about patients’ experiences of the healthcare system and the disability in low-income countries following this injury. The aim of the study was to explore patient experiences of treatment and disability following an open tibia fracture in Malawi.

**Methods: **A qualitative study was conducted using semi-structured interviews with ten patients with open tibia fractures at a central hospital in Blantyre, Malawi. A mixed deductive-inductive thematic analysis was used to identify broad themes of treatment and disability. Written informed consent was obtained from all participants.

**Results:** Patient characteristics included an average age of 39.1 years old (22-63) and 80% were male. Broad themes found were delays in receiving treatment, change in individuals’ societal role and delayed recovery associated with pain and immobility.

**Conclusions:** Open tibia fractures in Malawi have a devastating impact on patients and their families. Further studies are required to explore the reasons for the delays in open fracture emergency treatment.

## Introduction

The World Health Organization’s Global Status Report on road safety suggests that 90% of road fatalities occur in low- or middle-income countries (LMICs) in spite of having only 54% of the world’s vehicles ^1^. For each person that dies from trauma, up to ten become permanently disabled, many of whom could benefit from effective surgical interventions ^2^. Road traffic injuries (RTIs) are rapidly increasing in Africa due to high traffic volume, poor driver training, poor law enforcement, and vulnerable road users such as pedestrians and bicyclists ^3, 4^.

Open tibia fractures are one of the most common consequences following RTIs and a common cause of hospital admissions ^4^. In LMICs these injuries have devastating consequences, including an around 15% amputation rate, 18% infection rate, 20% non-union rate, and only 20% of patients with an open tibia fracture return to work after one year ^5^.

Quality of life (QOL) is defined as an individual's perception of their position in life affected in a complex way by the person's physical health, psychological state, personal beliefs, social relationships and their relationship to salient features of their environment ^6^. Previous qualitative studies from Uganda and Malawi ^7, 8^ showed the importance of social support as well as a large impact on quality of life from lower limb injury through reduced economic resources. Many QOL studies have not looked specifically at open fractures, despite this being identified as a priority by the Lancet Commission on Global Surgery ^9^. A qualitative study on lower limb open fractures from high-income countries suggests patients feel disempowered and changed ^10^, but due to the different setting, we hypothesised that patients’ experiences in LMICs are very different. Our aim was to explore patient experiences of treatment and disability following an open tibia fracture in Blantyre, Malawi.

## Methods

### Ethics

This study was reviewed and approved by the University of Malawi College of Medicine Research and Ethics Committee (P.07/19/2739). Written and verbal informed consent was obtained from all participants included in the study prior to starting the interviews.

### Setting: Queen Elizabeth Central Hospital, Blantyre, Malawi

Basic health care is provided by the government in Malawi free of charge at primary, secondary and tertiary hospitals. However, there are no emergency ambulance services currently in place in the country. About 45% of the fatally injured road users are pedestrians and close to 20% are cyclists where open fractures are becoming one of the most common causes of admission ^3^.

The government aims to have primary level facilities with outpatient clinics that can conduct basic procedures such as casting. Secondary level hospitals (district hospitals) should be able to treat open fractures with analgesia, antibiotics, debridement, internal and external fixation. Tertiary services (central hospitals) should be able to conduct the above, as well as more complex procedures such as flaps and bone transport. The above provision of care is not always possible due to limited resources in this setting ^11^. The Queen Elizabeth Central Hospital (Blantyre) has a capacity of 1000 beds and a catchment population of 7 million in southern Malawi. It has specialist services such as an intensive care unit and support from allied professionals such as physiotherapy. Surgeons usually review patients on weekly ward rounds due to limited availability from other commitments such as operative theatre and follow-up clinics.

### Participant selection

Purposive sampling was used to select our study sample and represent the population in Malawi. Open tibia fractures typically affect a homogenous population of young males with the main cause being from road traffic injuries ^4^. To understand common perceptions and experiences among a group of relatively homogeneous individuals, twelve interviews should suffice ^12^. Recent guidelines for thematic analysis ^13^ suggest 6–10 participants for small projects. We therefore approached participants face-face at the hospital and decided to conduct ten semi-structured interviews with participants who were 18 years or older.

### Data collection

Medical notes were reviewed to confirm an open tibia diagnosis and patient demographics. The interviews were conducted by WS, a male Chichewa speaking qualitative researcher with experience in qualitative research in tuberculosis and HIV ^14^, and in the presence of a male orthopaedic surgeon (AS) with knowledge of open fractures in LMICs. Patients were approached face-face at their bedside, and written and oral informed consent was obtained before conducting the interviews in a clinic room off the two orthopaedic wards.

In order to explore patients’ experiences of treatment and disability, the interview guide in English or Chichewa included questions about the injury, the treatment received, interactions with healthcare staff, home living situation, social interactions, employment, masculinity and perceived health and QOL (see question guide in Table 1). Audio recordings were transcribed verbatim and translated into English by one of the interviewers (WS). The mean length of interviews was 47 minutes (range 23–61 minutes). Field notes were taken by AS to document non-verbal expressions and WS’ initial thoughts.

**Table 1.** English interview guide.

**What is the understanding of disability for patients with an open tibia fractures in Malawi?** The interviews will be with the participant, the interviewer (research assistant) and an observer. They will be recorded and last 1hr–1hr30mins.
**Introduction** • Introduce self • As you are aware a research team are currently doing some research into open tibia fractures and its treatments in which you were involved. • We would like to ask you some questions about your experiences. • This will take about one hour. An information sheet about the study has already been given to you to read. Do you have any questions about the study? Before we continue with the interview are you still happy to participate? • Go through this and the consent form to ensure the participant is OK about participating, cover the points about audio recording, data protection, confidentiality and anonymity.
**Topic Guide** 1. We, Drs, understand you have had a serious accident, could you tell me how it has affected your life? * How would you describe your overall health now?* *Comparing yourself to before injury, are there any things that you struggle to do physically? Can you describe them.* *Comparing yourself to before injury, how do you find moving about?* 2. Do you have any fears with this injury? * Have you felt anxious/worried? If so when?* *Do you know anyone with this injury?* 3. Can you tell me about the treatment you received? * Was the treatment discussed with you/ family member?* *Did you decline any treatment that was offered to you? Why?* *Do you think it helped you? If so, in what way? If not, why not?* *How long were you in hospital? Was this long enough? Too long? Did you stay for the whole time that was suggested by the medical * *team? If not, why?* *Did you go back to hospital once you left? If not, why?* *What do you think of amputations?* 4. How has your mood been since the injury? * How is your sleep?* *Are you ever uncomfortable when lying/sitting down?* *When is the pain worse?* *How has this injury affected your family/friends?* *Have your relationships changed? In what way?* *Have they helped you? How?* 5. Does it affect your ability to work? * Are you working (any time spent in exchange for money or goods)?* *How quickly have you returned to work? What affected your decision (e.g. you felt better, you needed to work etc.)?* *How could you have gotten back to work quicker?* *Are you earning as much as before the injury? Why/ why not?* *Have you sold any possessions following injury? Why?* *Are you in pain at work? How do you cope?* 6. From your experience of the hospital, what do you think the hospital should be doing? * How could your treatment have been better? (more medication (which)? more time with doctors? Better explanations? Closer * *treatment to home? )* *What would you need most after hospital (once you leave)?* **7.** Is there anything else you want to tell us?
**Thank the participant for their valuable contribution and explain the next stage in the process of research and how their involvement will contribute.**

No repeat interviews were carried out. Transcripts were not returned to participants. No participants refused or dropped out of the study.

### Data analysis

Information from the medical notes was triangulated with interview transcripts. Thematic analysis, described by Braun & Clark ^15^ is widely used in psychology, healthcare research and social research. We conducted a mixed deductive-inductive thematic analysis as these complement each other.

Firstly, a template in the form of codes from a codebook ( Table 2) was used to organise text for subsequent interpretation according to the COREQ checklist ^16, 17^. The template was defined before commencing the analysis of the data and was developed using insights from the literature as well as the interview guide to organise it based on general quality of life categories. As this was an exploratory study, no a priori theoretical framework was used.

**Table 2.** Coding tree used in data analysis.

Codes	Subcodes
Source of injury	Car Mini-bus Motorcycle Cyclist Pedestrian Passenger Driver Commute to work Visit friend/family
Access to help	Friend Stranger Delay (before hospital) Distance to hospital District hospital Central hospital
Treatment in hospital	Delays (in hospital) Pain management Understanding of treatment Explanation of treatment Surgery Interaction with clinicians Interaction with nurses Length of hospital stay Medication Amputation Improved treatment Physiotherapy Prosthetics Rehabilitation Different treatment to others on the ward
Function	Walking Self-care (bathing) Eating Independence Immobility
Disability	Appearance Interaction with environment Embarrassment Stigma Disfigurement Coping mechanism Pain Pain medication Distraction
Mental health	Fear Depression Lack of sleep Poor appetite Tearful Anger Weight loss Anxiety
Relationship with friends and work	Dependants Change of family dynamics School Masculinity Reliance on others Loss of income Borrowing/loans Hunger

Transcripts were then imported into NVIVO 12 QRS software (QSR, Melbourne, Australia) for organisation, management and coding. These were deductively coded by carefully reading and re-reading transcripts, identifying emerging codes from the data by two coders (AS and WS). One of the coders had qualitative research experience and any disagreements in coding were discussed with senior qualitative authors (ND and MK).

Encoding the information organised the data to facilitate the identification and development of themes from them ^18^. We identified recurrent themes and patterns across groups by looking at code co-occurrences and frequencies, moving from specific instances to more broad concepts. The results were then reported according to the qualitative research COREQ checklist ^17^.

## Results

We conducted ten semi-structured interviews with participants who were 18 years or older. Demographic characteristics of study participants are provided in Table 3. We aimed to achieve a diverse spread of patients (age, grade, length of hospital stay). Time from injury varied from five days to one year after injury. Some participants were sent home and re-admitted for further procedures. Nine out of ten patients who participated in this study suffered road traffic injuries with the mechanism being pedestrian vs car. Most were either at work during injury or commuting to work. Eight out of ten participants of our study were male. Participants’ average age was 39.1 years old (22-63). Occupation and education levels varied, but farmers were the most common with primary school level education. Eight out of ten participants were from rural areas while two were from urban Blantyre. Eight out of ten were married. Nine out of ten were referred to the central hospital from a district hospital or healthcare centre. Interviews were conducted on average 68 (5-368) days after injury. Most patients who participated in this study had a Gustilo-Anderson Grade III fracture. They received an average of 1.8 (0-5) operations such as debridement, external fixators, SIGN nail, split skin grafts and bone transport. No patient had managed to return to work after their injury.

**Table 3.** Characteristics of open tibia fracture study sample.

	Number
**Gender**	
Male	8
Female	2
**Age**	Mean 39.1 years (SD=10.6)
**Method of injury**	
Pedestrian-vehicle trauma	8
Single-vehicle trauma	1
House injury	1
**Relationship status**	
Married	8
Widowed	1
Single	1
**Education**	
Primary	5
Secondary	4
Tertiary	1
**House location**	
Rural	8
Urban	2
**Occupation**	
Small business owner	3
Farmer	2
Driver	2
Labourer	1
Public servant	1
Housemaid	1
**Gustilo Anderson grade of ** **fracture**	
Grade I	2
Grade II	3
Grade III	5
**Initial treatment centre**	
Healthcare centre	3
District hospital	6
Central hospital	1
**Number of operations**	Mean 1.8 (SD=1.8)
**Time from interview to injury**	Mean 68 days (SD=118)

### Overarching themes

Disability is a complex phenomenon with interactions between a person’s body and features in society in which they lives ^6^. Results from the analysis of qualitative data collected in this study demonstrate three key emerging themes, namely 1) delayed treatment, 2) change in societal functions, 3) slow recovery.

Theme 1: Delayed treatment following injury

*1) Access to treatment*

On the health service demand side, there was no delay in seeking medical attention as participants felt they needed medical help urgently due to pain, lack of mobility and visual deformity of their bone being exposed. Most participants received first aid and transport to healthcare facilities either from a bystander or a family member.

*“It is very obvious that my leg has to be taken to the theatre” P9, 40 year old female, Grade I*

Nine out ten participants presented to a healthcare centre or district hospital before being referred to the central hospital. In district hospitals, they felt they received no or limited treatment; they described it as basic care and felt they had to wait a long time for this. We did not ask participants to estimate how far they came from or how long the delay was. Transportation issues were a common problem for referrals between district hospitals in southern Malawi and central hospitals. Due to low funding to the district hospitals in Malawi, ambulances travelling to a referral tertiary hospital usually wait until they have an adequate number of patients to transport to a central hospital.

*“If it (leg) stays long here it means as they have dressed it, they haven’t cleaned it and left me in the ward” P2, 39 year old male, Grade IIIB*

*2) Trust in doctors*

Some participants praised the doctors and described them as dedicated, dealing with difficult circumstances and limited resources that contributed to delays. They described the instances of contact with doctors as their weekly highlight, as more decisions were made on these days. They did not extend this same praise to other allied professionals.

*“…your leg has been extensively damaged and we can’t manage treating it here you are supposed to go and meet a white doctor in Blantyre so that he can put some metal…” P9, 40 year old female, Grade I*

*3) Patients as experts: Understanding of injury after hospitalisation and treatment*

Most participants were able to clearly articulate their injury and the treatment received. They were able to describe the difference between internal and external fixations and described procedures such as skin grafts and bone lengthening. They were also aware of the risk of infection and its links with delayed operations and risk of amputation. Their understanding mainly came from doctors’ explanations as well as seeing their limb exposed through skin. Urban or rural location and education of the participants did not influence understanding.

*“When they come to collect pus for laboratory tests that is when they find more bacteria and that lengthens the days I am here.” P4, 44 year old male, Grade IIIB*

Theme 2: Change in societal position and function

The subsequent two overarching themes reflected that disability is a complex phenomenon with interactions between a person’s body and features in society in which they lives ^6^. The second theme underlined a shift in participants’ roles in society from supporting their families to being dependant on their family for food, finances and basic care. It also highlighted amplified levels of social stigma directed towards recovering patients.

*1) A shift from breadwinners to beggars*

Many participants mentioned that prior to injury they had many dependants (e.g. spouse, children etc.) along with the responsibility to provide for them. Their parental responsibilities included paying for their children’s school fees, providing shelter, clothing and food. Whilst an inpatient, they often felt guilty and anxious about their dependents at home without having someone to fend for them. Study participants’ fear of financial loss was mainly emanating from loss of potential income whilst being an inpatient in hospital. Upon discharge, they had all considered alternative sources of income different from their occupation before the injury, but realised that their injury prevented them from working mainly due to reduced mobility and pain.

*“Right now they [children] were the ones that were depending on me, but now it is them that go look for money to help me, that’s where I see that I am a burden to them” P2, 39 year old male, Grade IIIB*

*2) Friend/family support*

Support from family and friends was considered very important for the recovery of all our participants. This also had wider consequences on a societal level. Firstly, participants reported that their guardians, (people who had to care for inpatients), found it difficult to care for children at home as they had to relocate from their own home of residence, sometimes long distances, in order to care for their injured loved ones. Some described reduced social contact partly due to the long distance of the central hospital from their hometown. Friends felt like they needed to give money and food to the injured. Some of the participants became tearful during the interviews when talking about the breakdown of family relations and support.

*“… some of my relatives have got their own families but they have left them and they are taking care of me here.” P9, 40 year old female, Grade I*

*3) Social stigma*

There was also significant perceived social and self-stigma associated with being disabled or having an amputation emanating from an open tibia. This is due to the incapacity to provide for their family caused by this disability. Older participants felt they had lost their social position and no longer received community privileges such as the community bank or limited job opportunities. Many also reported being ridiculed overtly by other members of the community or feeling their views were considered less important immediately following injury.

*“For example those that are disabled are insulted and called all sorts of names, but they forget it is an accident.” P9, 39 year old male, Grade I*

Theme 3: Slow recovery

Study participants displayed hope that they would eventually recover, but slow recovery was associated with physical morbidity and pain.

*1) Hope*

Many of the participants were hopeful they would eventually make a full recovery from the injury. Their hope for a full recovery came from many sources, including family and friend support, seeing other people with similar injuries recover, understanding their injury and treatment, interactions with senior healthcare staff, resilience and religion.

*“First of all my hope comes from God and secondly the doctors. In terms of God I believe in him [God] because he is the controller of everything. The doctors as well they are the ones that fix everything like treating the broken bones, for instance the way it is here they are the ones that will lengthen my leg.” P10, 22 year old male, Grade IIIB*

*2) Pain*

Participants often associated severity of injury with amount of pain when asked to compare their injury to others. Some participants experienced severe pain when having debridement without general anaesthesia. This usually occurred in district hospitals and happened because of lack of resources and operative capacity. They associated pain severity with death as described by a male participant when he was undergoing debridement procedure.

*“Everywhere in my body there was severe pain to the extent that I thought I am losing my life.” P3, 32 year old male, Grade II*

*3) Walking*

Walking is associated with recovery and better quality of life. Participants mentioned walking improved following their injury and treatment. Walking was perceived to be crucial for participants to be able to return to work. They described it being disheartening to witness friends walk and being discharged whilst they remained immobile for prolonged periods of time. Walking was also noted as causing severe pain. Participants also perceived normality as being able to walk as they did prior to their injury.

*“Recovery to me means being able to walk properly like before.” P10, 22 year old male, Grade IIIB*

## Discussion

This is the first study to describe patients’ experiences of an open tibia fracture in a low-income country. Participants experienced poor outcomes following an open tibia fracture, including shame, guilt, pain and immobility. This was made worse by delayed treatment and improved by gaining an understanding of the injury, trust in doctors and hope. The trust in doctors might have been influenced by one interviewee being an orthopaedic surgeon, however, participants were still comfortable discussing delays. The negative impact on quality of life is similar to other qualitative studies on patients with lower-limb injuries reported in other low- or middle-income countries ^7, 8^.

Referral to tertiary facilities to receive appropriate surgical care was a major hurdle for our participants. Current evidence suggests that complex open fractures (Grade III) and some of their complications should be managed in specialist central hospitals, as delays in debridement and soft tissue coverage are known to be associated with higher infection, non-union and amputation rates ^5^. In Malawi, better trauma networks and standardisation of care are required to minimise patients’ experiences of delay to open fracture treatment ^19^. This may also be improved by increasing surgical capacity or surgical and anaesthetic training ^20^. It is unclear how the variability of treatment in district hospitals impacts patients’ perspectives. Further studies should explore opinions from district level management and healthcare providers including orthopaedic staff as to the reasons of referral delays in Malawi. This should also include reasons for inadequate anaesthesia during a debridement.

Malawian public health services are free and, therefore, patients did not express any fear over cost of treatment, but they were worried about their potential loss of income whilst in hospital. Financial loss was fundamental to all our participants (whether male or female), who felt guilty about not being able to maintain usual family responsibilities ^10^. Studies suggest that only 30% of patients return to work at one year following a lower limb injury in Uganda ^21^. Other studies on lower limb injuries have suggested large indirect costs to patients and their guardians from guardians taking care of injured patients, food costs and transport costs ^22^. Further studies are required to assess the detailed economic evaluation of this injury to patients, which is a key global indicator of the Lancet Commission ^9^.

Alleviating pain and improving walking was the key to recovery following a lower limb injury, as in high-income countries ^10^. Immobility and pain contributed to reduced ability to perform daily activities such as going to the toilet, which has also been reported in other studies ^7, 23^. As all participants described walking and pain to be key to recovery, further research needs to be conducted in outcomes of open fractures that include pain and walking. As participants described ongoing pain and immobility post discharge from hospital, further studies could explore programmes for re-integration back into the community.

Our sample size and study setting may limit the generalisation of the results. The participants treated solely in district hospitals may have different experiences from those patients referred to a central hospital from district hospitals (due to the limited resources and expertise which have a bearing on the desired clinical outcome). This was, however, the first qualitative study of its kind and a first exploration of patients’ experiences. The qualitative approach facilitated a depth of understanding of disability and treatment and the context of participants with this injury. We did not recruit any children to our study, but other studies on children with injuries found similar themes including stigma, pain and hunger ^24^. A strength of this study was that we included open tibia fractures from varied lengths after injury (five days up to one year). Other studies in high-income countries have shown that patients with open fractures still have disability with similar themes, such as slow recovery, lack of independence and immobility, even after four years ^10^. Further long-term qualitative and quantitative studies of open fractures are required in order to understand the lasting consequences for patients.

## Conclusion

In conclusion, patients expressed guilt, shame, pain, immobility and frustrations with delays following an open fracture treatment. This may be improved with more integrated trauma networks, increased surgical and anaesthetic capacity and training, more social support and walking aids. More work is needed to quantify patients’ long term function, pain and fear of financial loss.

## Data availability

### Underlying data

Interview recordings and transcripts cannot be shared due to patient confidentiality. As per the protocol that the ethics committee approved, data can only be shared with members of the study team. The interview brief is available in Table 1, the coding tree in Table 2, and quotes representing the transcripts are available in the manuscript.
