# Supplementary material for: “Don’t rush into thinking of walking again”: Patient views of treatment and disability following an open tibia fracture in Malawi
Source: Wellcome Open Res. 2022 Aug 9;7:204. [Version 1] doi: 10.12688/wellcomeopenres.18063.1 (PMC9453110; doi:10.12688/wellcomeopenres.18063.1)
Supplement: Peer review history

# Peer review: Reviewer response for version 1

**DOI:** 10.21956/wellcomeopenres.20031.r51976

**Published:** 2022-09-07

**Authors:** Chris Lavy

**Affiliations:** 1 Nuffield Department of Orthopaedics, Rheumatology and Musculoskeletal Sciences, Botnar Research Centre, University of Oxford, Oxford, UK

This paper is a qualitative study of 10 patients with open tibial fractures. This is a common injury as the tibia is so close to the skin and there are many factors affecting healing.  A semi-structured interview was conducted with each patient and this was analysed using NVIVO which is an industry standard software for qualitative work. The results were unsurprising in some aspects, ie the patients were mainly young males and the accidents involved motor vehicles. What was new in the literature, at any rate I have not seen it so clearly outlined, is the very poor outcome, with none of the patients having returned to work. The other striking and sad finding is the social aspect, the effect on family and kin, the effect on finances and relationships. It is clear that this injury is devastating, and it is also clear that more research needs to be done in this area.  

 It was not clear exactly how they authors chose the participants.  They used purposive sampling but do not give details.  A small addition would help here.

 I think that this paper is a very important addition to the literature, and merits indexing. I think and hope that it will encourage more work in this area.

Is the work clearly and accurately presented and does it cite the current literature?

Yes

If applicable, is the statistical analysis and its interpretation appropriate?

Not applicable

Are all the source data underlying the results available to ensure full reproducibility?

No

Is the study design appropriate and is the work technically sound?

Yes

Are the conclusions drawn adequately supported by the results?

Yes

Are sufficient details of methods and analysis provided to allow replication by others?

Yes

Reviewer Expertise:

Orthopaedics, trauma, spine surgery, infection, clubfoot, LMIC surgery, global surgical education

I confirm that I have read this submission and believe that I have an appropriate level of expertise to confirm that it is of an acceptable scientific standard.

# Peer review: Reviewer response for version 1

**DOI:** 10.21956/wellcomeopenres.20031.r51977

**Published:** 2022-08-26

**Authors:** Dominic Konadu-Yeboah

**Affiliations:** 1 Directorate of Trauma and Orthopaedics, Komfo Anokye Teaching Hospital, Kumasi, Ghana

**Article Summary**

 This is the first qualitative study that explores patients' experiences on open tibial fractures in a low income country. The study includes open fractures of the tibia of varying duration from 5 days to 1 year in district hospitals.

 The cardinal themes reported are delays in receiving treatment, alteration of patients' societal roles and protracted recovery from the injury due to pain and immobility. 

**General comment**

 The study carries the potential of contributing to knowledge in the field of open tibial fracture treatment particularly in assessing the quality of life of patients after the injury and its treatment.

 There are a few sentence construction errors to address:

 Overarching Themes

1. Theme number 3): "....... in which they lives
  Comment: This should be " .... in which they live
2. Patients "had pain when having debridement without general anaesthesia"
  Comment: Specify the mode of anaesthesia that these patients received. Did some undergo spinal anaesthesia and yet perceived pain?

Data collection

 Time from injury varied from 5 days to 1year and the patients were interviewed on the ward.

 Comment: Does this seek to imply that some of the patients stayed on the hospital ward for 1 year or did some of the interviews take place in the community? This should be clarified.

 Results

1. We aimed to achieve ........ (age, grade, length of hospital stay)
  Comment: "grade"  should be " grade of injury"
2. Farmers were the most common ...
  Comment: Should be  "Farming was the most common occupation"
3. Most of the patients were rural farmers who sustained the injury while at work or while commuting to work and nine out of ten sustained road traffic injuries.
  Comment: Were these farmers commuting on foot or in a vehicle at the time of the road traffic accident or did they sustain the injury on the farm?

Addressing these few issues will improve the quality of the paper.

Is the work clearly and accurately presented and does it cite the current literature?

Yes

If applicable, is the statistical analysis and its interpretation appropriate?

Yes

Are all the source data underlying the results available to ensure full reproducibility?

Yes

Is the study design appropriate and is the work technically sound?

Yes

Are the conclusions drawn adequately supported by the results?

Yes

Are sufficient details of methods and analysis provided to allow replication by others?

Yes

Reviewer Expertise:

Musculoskeletal trauma

I confirm that I have read this submission and believe that I have an appropriate level of expertise to confirm that it is of an acceptable scientific standard.
